# The Role of E3 Ubiquitin Ligase Cbl Proteins in Interleukin-2-Induced Jurkat T-Cell Activation

**DOI:** 10.1155/2013/430861

**Published:** 2013-03-27

**Authors:** Ming-Fang Zhao, Xiu-Juan Qu, Jing-Lei Qu, You-Hong Jiang, Ye Zhang, Ke-Zuo Hou, Hao Deng, Yun-Peng Liu

**Affiliations:** ^1^Department of Medical Oncology, The First Hospital of China Medical University, Shenyang 110001, China; ^2^Institute of Cancer Research, The First Hospital of China Medical University, Shenyang 110001, China

## Abstract

Interleukin- (IL-) 2 is the major growth factor for T-cell activation and proliferation. IL-2 has multiple functions in the regulation of immunological processes. Although most studies focus on T-cell immunomodulation, T-cell activation by IL-2 is the foundation of priming the feedback loop. Here, we investigated the effect of MAPK/ERK and PI3K/Akt signaling pathways on IL-2-induced cell activation and the regulatory mechanisms of upstream ubiquitin ligase Cbl-b and c-Cbl. Morphological analysis of Jurkat T cells was performed by cytospin preparations with Wright-Giemsa stain. CD25 expression on Jurkat T cells was determined by flow cytometry. Changes in cell activation proteins such as p-ERK, ERK, p-Akt, Akt, and ubiquitin ligase Casitas B-cell Lymphoma (Cbl) proteins were analyzed by western blot. Following IL-2-induced activation of Jurkat T cells, p-ERK expression was upregulated, while there was no change in p-Akt, ERK, or Akt expression. Thus, the MAPK/ERK signaling pathway, but not PI3K/Akt, was involved in IL-2-induced T-cell activation. Either using PD98059 (a specific inhibitor for p-ERK) or depletion of ERK with small interfering RNA (siRNA) reduced the expression of CD25. This study also showed that ubiquitin ligase proteins Cbl-b and c-Cbl might be involved in IL-2-induced Jurkat T-cell activation by negatively regulating the MAPK/ERK signaling pathway.

## 1. Introduction

In the late 1970s, Smith et al. first described interleukin- (IL-) 2 as a T-cell growth factor produced by T lymphocytes [1]. Further studies demonstrated that IL-2 was the major growth factor for T-cell activation and proliferation. IL-2 signaling occurs via the IL-2 receptor (IL-2R) that consists of the IL-2R alpha (CD25), IL-2/IL-15R beta (CD122), and common gamma (gc; CD132) chains [2]. IL-2R signaling is initiated by phosphorylation of JAK3 and JAK1, which are constitutively associated with the gc and IL-2R beta chains, respectively. Activation of these kinases leads to the activation of PI3K/Akt, MAPK/ERK, and the STAT family of transcription factors [3]. However, the signaling pathways upstream of the T-cell receptor (TCR) network are unclear.

The ubiquitination of proteins by E3 ligases is an important regulatory mechanism for a variety of immune functions, such as maintenance of T-cell homeostasis and self-tolerance [4, 5]. Cbl-b was the first E3 ubiquitin ligase to be directly implicated in T-cell activation and tolerance *in vivo* [6, 7]. Casitas B-cell Lymphoma (Cbl)-b belongs to a highly conserved family of proteins, which in mammals consists of three homologues: c-Cbl, Cbl-b, and Cbl-3 [8]. Cbl proteins downregulate multiple signaling pathways by ubiquitylating receptor tyrosine kinases, thereby targeting them for degradation. Among these proteins, Cbl proteins can interact with the p85-regulatory subunit of phosphoinositide 3-kinase (PI3K), which leads to PI3K ubiquitination and degradation [9, 10]. PI3K catalyzes the production of phosphatidylinositol-3,4,5-trisphosphate, activates the downstream Akt, and therefore contributes to the activation of various signaling components involved in the regulation of gene expression and cell survival [11]. Several studies have shown that T cells expressing active Akt are resistant to activation-induced apoptosis *in vitro* and *in vivo* [12, 13]. However, it remains unknown whether Cbl-b and c-Cbl are involved in the activation of T cells, through downregulation of the PI3K/Akt signaling pathways.

Other pathways including the mitogen-activated protein kinase (MAPK) superfamily, ERK, JNK, and p38 MAPK, are also important regulators for IL-2 gene transcription. Both ERK and JNK activities were stimulated upon anti-CD3/CD28 ligation [14]. CTLA-4, also known as CD152, also inhibits TCR-induced ERK and JNK activation [15]. c-Cbl ubiquitin ligase can also regulate MAPK/ERK activity [16, 17]. Recently, we showed that c-Cbl mediated an inhibitory effect on TRAIL-induced apoptosis through the downstream MAPK/ERK signaling pathway [18]. However, the potential role of the c-Cbl-dependent MAPK/ERK pathway in the activation of T cells has not been identified.

To investigate the mechanisms of IL-2-induced T-cell activation, we used a human leukemia cell line, Jurkat T cells, as a model to study the expression of a cell surface activation marker CD25, the effect of MAPK/ERK and PI3K/Akt signaling pathways in the activation process, and the regulatory mechanisms of upstream ubiquitin ligase Cbl-b and c-Cbl.

## 2. Results and Discussion

### 2.1. IL-2-Induced Jurkat T-cell Activation

**Figure 1:**
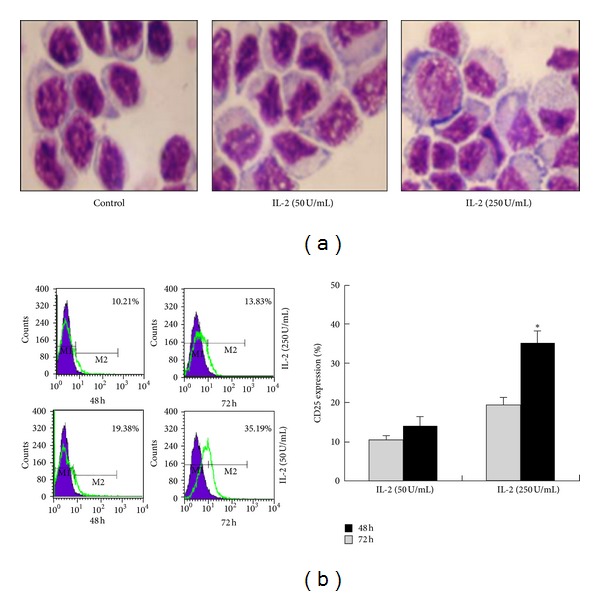
IL-2 induces Jurkat T-cell activation. (a) Morphological analysis was performed by cytospin preparations of T cells and Wright-Giemsa stain. (b) Jurkat T cells were exposed to different concentrations of IL-2 (50 U/mL or 250 U/mL) for 48 h or 72 h. CD25 expression was measured by flow cytometry. Mean ± SE (*n* = 3). *P < 0.05.

To verify whether IL-2 induces T-cell activation, Jurkat T cells were exposed to different concentrations of IL-2 (50 U/mL and 250 U/mL) for 48 h or 72 h. Morphological analysis revealed that most cells increased in size, had abundant cytoplasm, vacuoles in the cytoplasm, loose nuclear chromatin, an obvious nucleolus, and resembled lymphocytoblasts (Figure 1(a)). Following incubation of Jurkat T cells with IL-2, cells were stained for CD25 and analyzed by flow cytometry. IL-2 at a concentration of 250 U/mL was the most effective concentration for activation as measured by maximal CD25 expression at 72 h (Figure 1(b)).

### 2.2. MAPK/ERK Signaling Pathway, But Not PI3K/Akt, Is Involved in IL-2-Induced Jurkat T-Cell Activation

**Figure 2:**
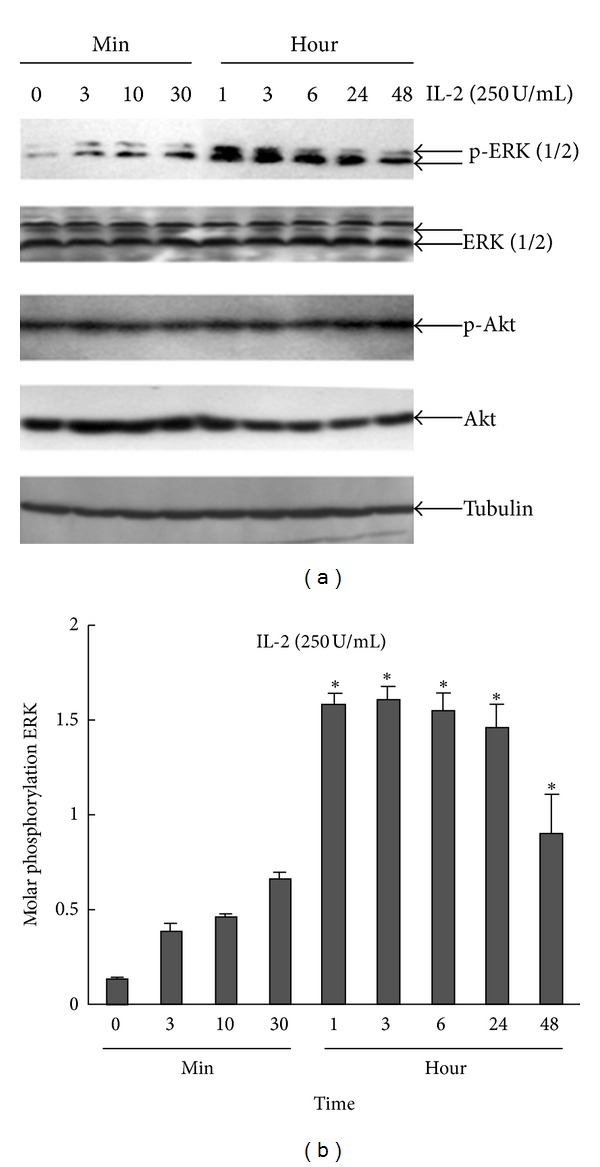
The expressions of p-ERK, ERK, p-Akt, Akt, and tubulin were measured by western blot. (a) Jurkat T cells were treated with IL-2 (250 U/mL) and harvested at the indicated time points. Lysates (50 *μ*g) were run on 10% SDS-PAGE followed by immunoblotting with p-ERK, ERK, p-Akt, Akt, and tubulin antibody. (b) The intensity of p-ERK for IL-2 treatment was estimated using NIH Image software as in (a) Mean ± SE (*n* = 3) from three separate experiments are presented.

To determine whether the MAPK/ERK signaling pathway is involved in activation of Jurkat T cells after exposure to 250 U/mL, IL-2 proteins from the signaling pathways were measured. Phospho(p)-ERK protein expression was upregulated quickly, with peak expression at 1 h, while there was no change in ERK expression (Figures 2(a) and 2(b)).

**Figure 3:**
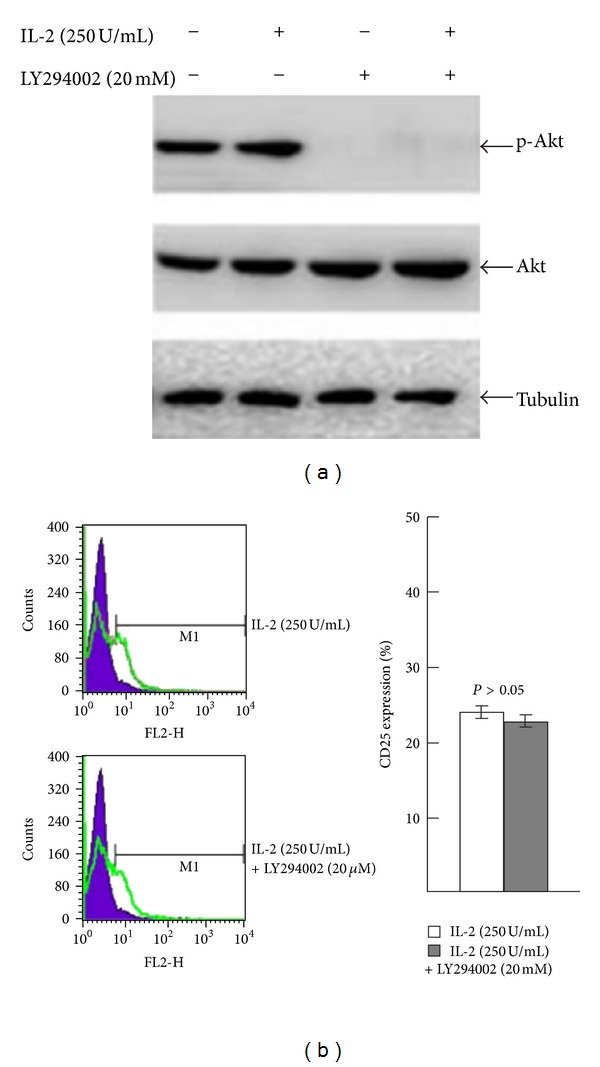
Jurkat T cells were exposed to IL-2 (250 U/mL) after using 20 mM LY294002. (a) After using 20 mM LY294002, p-Akt and Akt levels were measured by western blot. (b) CD25 expression was measured by flow cytometry. Mean ± SE (*n* = 3).

**Figure 4:**
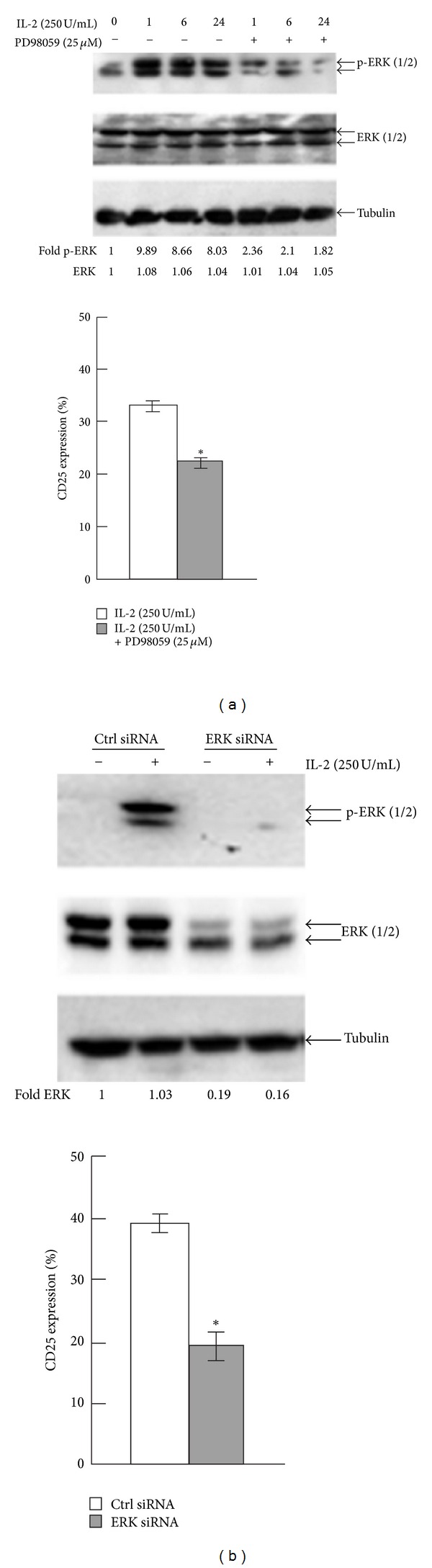
Jurkat T cells were exposed to IL-2 (250 U/mL) after using 25 *μ*M PD98059 or depletion of ERK with small interfering RNA (siRNA). (a) after using 25 *μ*M PD98059, p-ERK and ERK levels were measured by western blot. CD25 expression was measured by flow cytometry. (b) after depletion of ERK with small interfering RNA (siRNA), p-ERK and ERK levels were measured by western blot. CD25 expression was measured by flow cytometry. Mean ± S.E. (*n* = 3). *, *P* < 0.05.

To determine whether the PI3K/Akt signaling pathway was involved in IL-2-induced Jurkat T-cell activation, we measured p-Akt or total Akt expression by western blot. IL-2 (250 U/mL) did not affect either p-Akt or total Akt protein levels. Pretreatment of Jurkat T cells with the PI3K-specific inhibitor, LY294002 (20 mmol/L), had no significant effect on the expression of CD25 (Figures 3(a) and 3(b)). Taken together, these results indicate that the MAPK/ERK signaling pathway, but not the PI3K/Akt signaling pathway, plays a crucial role in regulating IL-2-induced Jurkat T-cell activation. We next investigated whether the presence of PD98059 (a specific inhibitor for p-ERK), or depletion of ERK with small interfering RNA (siRNA), could affect IL-2-induced Jurkat T-cell activation. Both using PD98059 (25 *μ*M) for 30 min and ERK siRNA significantly reduced the expression of p-ERK. And the expression of CD25 was decreased from 32.98% to 22.32% (PD98059) and from 39.23% to 19.31% (ERK siRNA) (*P* < 0.05) (Figures 4(a) and 4(b)). Thus, ERK was involved in the process of IL-2-induced T-cell activation at the protein level.

### 2.3. Cbl-b and c-Cbl Negatively Regulate ERK Signaling Pathways during IL-2-Induced Jurkat T-Cell Activation

**Figure 5:**
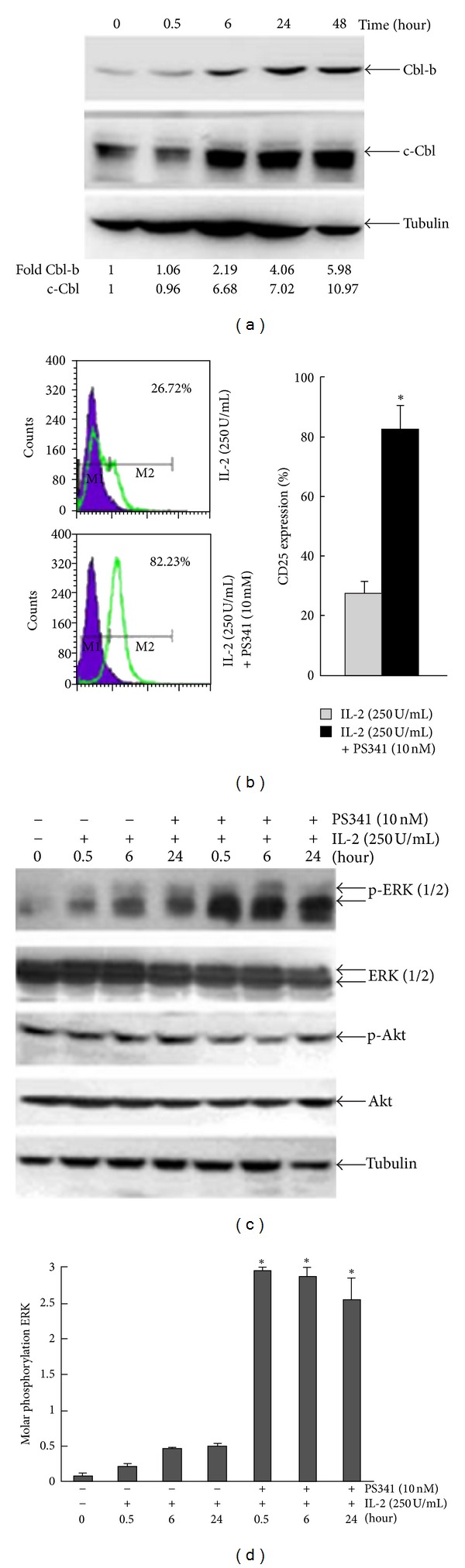
Effects of IL-2 (250 U/mL) on upregulating the expressions of c-Cbl and Cbl-b in Jurkat T cells. (a) The expression levels of Cbl-b and c-Cbl were measured by western blot in Jurkat T cells treated with 250 U/mL IL-2 for 48 h. (b) Jurkat T cells were exposed to IL-2 (250 U/mL) in the absence or presence of 10 nM PS341, and CD25 expression was measured by flow cytometry. (c) After exposure to 250 U/mL IL-2, the expressions of p-ERK, ERK, p-Akt, Akt, and tubulin were measured by western blot in the presence or absence of 10 nM PS341. (d) The intensity of p-ERK for IL-2 and/or PS341 treatment was estimated using NIH Image software as in (c) Mean ± SE (*n* = 3) from three separate experiments are presented.

Whether the Cbl family of ubiquitin ligases inhibited ERK during T-cell activation by IL-2 is unknown. As shown in Figure 5(a), the expression levels of Cbl-b and c-Cbl of Jurkat T cells treated with 250 U/mL IL-2 for 48 h reduced gradually to a minimum level at 24 h. In general, this was coincident to the change of proteins associated with activation. The results indicated that Cbl proteins are involved in the IL-2-induced activation process and possibly are upstream of the ERK signaling pathways. To confirm the role of Cbl proteins, we pretreated Jurkat T cells for 30 min with PS341, a proteasome inhibitor that suppresses the functions of Cbl proteins, followed by incubation with IL-2, and observed an increase in CD25 expression at 72 h compared with control cells. This indicated that PS341 could enhance CD25 expression and confirmed that Cbl proteins are indirectly involved in IL-2-induced Jurkat T-cell activation (Figure 5(b)). The expression of p-ERK at 0.5 h, 6 h, and 24 h in PS341 pretreatment group measured by western blot was greater than in the untreated control group, suggesting that Cbl proteins are involved in IL-2-induced Jurkat T-cell activation (Figures 5(c) and 5(d)).

### 2.4. Discussion

To investigate the mechanisms of IL-2-induced T-cell activation, we used Jurkat T cells to study the change in cell surface expression of CD25, a marker of cell activation. In addition, the role of MAPK/ERK and PI3K/Akt signaling pathways in process cell activation and the regulatory mechanisms of upstream ubiquitin ligase Cbl-b and c-Cbl were studied. Here, we report that the MAPK/ERK signaling pathway, but not PI3K/Akt pathway, was involved in IL-2-induced T-cell activation. Furthermore, our data show that Cbl-b and c-Cbl were involved by negatively regulating the ERK signaling pathway.

Previous studies indicated that IL-2 exerts complex immunological functions to promote the proliferation, survival, and activation of T cells and induce immune regulatory mechanisms [19]. Thus, IL-2 is used clinically to generate specific immune responses and is inhibited to block unwanted responses. Recently, it has been suggested that IL-2 increases the frequency of regulatory T cells (Tregs) in cancer patients [20], and clinical studies in cancer patients have shown that treatment with recombinant IL-2 can induce the expansion of Tregs in peripheral blood [21–23]. Some of these studies were performed in young adult patients with sarcoma who were lymphopenic due to previous chemotherapy. Although most studies focus on the immunomodulation of T cells by IL-2, the mechanism of T-cell activation by IL-2 is still unclear.

IL-2R signaling is primarily mediated through activation of JAK1 and JAK3 with subsequent phosphorylation and activation of STAT3 and STAT5 [24]. These activated transcription factors translocate to the nucleus where they initiate a complex series of transcriptional events leading to many of the functional effects of IL-2 stimulation. PI3-K/AKT signal pathway is one of the important pathways which are involved in IL-family-induced activation. Cahill and Rogers reported that interleukin-1*β* induction of IL-6 is mediated by a novel phosphatidylinositol 3-kinase-dependent AKT/I*κ*B kinase alpha pathway targeting activator protein-1 [25]. IL-2R triggering also leads to the activation of other signaling pathways including MAPK and PI3K, which also contribute to the numerous functional effects of IL-2 on immune cells. However, other studies have reported that IL-2 primarily induces JAK/STAT signaling rather than PI3K signaling, and this may explain some of the differential effects of IL-2 on Tregs and effector T cells [26, 27]. In the present study, the results showed that PI3K/Akt signaling pathway did not appear to be crucial for the regulation of IL-2-induced Jurkat T-cell activation. However, the p-ERK protein expression was upregulated after exposure to 250 U/mL IL-2, and was inhibited by the presence of PD98059 (a specific inhibitor for p-ERK), which affected IL-2-induced Jurkat T-cell activation, suggesting that ERK signaling at the protein level is important. Pretreatment with PD98059 and incubation with IL-2 reduced the expression of CD25. Meanwhile, depletion of ERK with small interfering RNA (siRNA) significantly reduced the expression of CD25. These results indicated that it is MAPK/ERK pathway, but not PI3-K/AKT pathway, which was involved in IL-2-induced CD25 upregulation.

Proteasome degradation of ubiquitin-targeted proteins is an important mechanism that negatively controls activated signaling pathways [28]. The c-Cbl ubiquitin ligase can regulate MAPK/ERK activity [29, 30]. More recently, we showed that IFN-*α* strongly decreased c-Cbl protein expression, which paralleled an increase in p-ERK expression. Overexpression of c-Cbl attenuated IFN-*α*-induced ERK activation [18]. We investigated whether inhibition of ERK activation by IL-2 correlated with Cbl protein expression and observed that the expression levels of Cbl-b and c-Cbl reduced gradually. Pretreatment of Jurkat T cells with proteasome inhibitor PS341 (a suppressor of Cbl proteins) followed by exposure to IL-2 increased the expression of CD25 at 72 h compared to untreated control cells. In addition, the expression of p-ERK at 6 h and 24 h in the PS341 pretreatment group was further upregulated.

## 3. Experimental Section 

### 3.1. Reagents and Antibodies

Recombinant human IL-2 (Amgen, Thousand Oaks, CA, USA) was added to cultures at a concentration of 100 U/mL. Anti-phospho-ERK, anti-ERK, antitubulin, and anti-Cbl-b antibodies were purchased from Santa Cruz Biotechnology (Santa Cruz, CA, USA). Anti-p-Akt (Ser-473) and anti-Akt antibodies were purchased from Cell Signaling Technology (Beverly, MA, USA). Anti-c-Cbl antibody was purchased from Transduction Laboratories (Lexington, KY, USA). LY294002 was purchased from Sigma-Aldrich (St. Louis, MO, USA). PD98059 was purchased from Promega (Madison, WI, USA). The proteasome inhibitor PS-341 was purchased from Ben Venue Laboratories Inc. (Bedford, OH, USA).

### 3.2. Cells and Cell Culture

Jurkat T cells were obtained from the American Type Culture Collection (ATCC, Rockville, MD, USA). Jurkat T cells were cultured in RPMI 1640 medium that contained 10% heat-inactivated fetal bovine serum (FBS), penicillin (5 U/mL), and streptomycin (50 mg/mL) in a 95% air/5% CO_2_ atmosphere.

### 3.3. Morphology Staining

Cells were seeded at 2.5 × 10^4^ cells/well in 6-well plates and incubated overnight and then exposed to 50 and 250 U/mL IL-2 for 72 h. Morphological analysis was performed by cytospin preparations using the Wright-Giemsa stain (Sigma).

### 3.4. Small Interfering RNA Transfections

ERK small interfering RNA (siRNA) was obtained from Shanghai GeneChem Co. Ltd. (China). ERK siRNA was synthesised: 5′-GUGCUCUGCUUAUGAUAAUTT-3′. The Ctrl siRNA was synthesised: 5′-AATTCTCCGAACGTGTCACGT-3′. To silence *ERK* mRNA expression, we electroporated Jurkat cells with ERK siRNA. 300 nM of siERK or the transfection control (siRNA) were introduced into Jurkat T cells with Cell Line Nucleofector Kit V Solution Box (Amaxa, Lonza Cologne GmbH), following the supplier's instructions, and using the Nucleofector II (Amaxa, Lonza Cologne GmbH) electroporator. After transfection, Jurkat T cells were cultured in complete medium for 18 h prior to further experiment.

### 3.5. Western Blot

Cells were washed twice with ice-cold PBS and solubilized in 1% Triton lysis buffer (1% Triton X-100, 50 mmol/L Tris-Cl, pH 7.4, 150 mmol/L NaCl, 10 mmol/L EDTA, 100 mmol/L NaF, 1 mmol/L Na_3_VO_4_, 1 mmol/L phenylmethanesulfonyl fluoride, and 2 mg/mL aprotinin) on ice then quantified by the Lowry method. Samples of 50 mg of cell lysates were separated by SDS-polyacrylamide gel electrophoresis and electrophoretically transferred to nitrocellulose membranes (Immobilon-P, Millipore, Bedford, MA, USA). The membranes were blocked with 5% skim milk in Tris-buffered saline Tween-20 (TBST) buffer (10 mmol/L Tris, pH 7.4, 150 mmol/L NaCl, and 0.1% Tween-20) at room temperature for 2 h and incubated at 4°C overnight with the indicated primary antibodies. After washing with TBST buffer, membranes were incubated with the appropriate horseradish peroxidase-conjugated secondary antibodies for 30 min at room temperature. After extensive washing with TBST buffer, proteins were visualized using the enhanced chemiluminescence reagent (SuperSignal Western Pico Chemiluminescent Substrate; Pierce, Rockford, IL, USA) and signals were quantitated using NIH Image J software.

### 3.6. Statistical Analysis

Data are presented as means ± standard deviation. The significance of the difference between the groups was assessed by analysis of ANOVA then multiple comparisons were accounted for with Bonferroni. A *P* value of less than 0.05 was considered statistically significant. All means were calculated from at least three independent experiments.

## 4. Conclusions

In summary, the present study demonstrates that activation of Jurkat T cells induced by IL-2 is mediated by enhanced activation of the MAPK/ERK pathway, which influences the expression of CD25. Furthermore, ubiquitin ligase proteins Cbl-b and c-Cbl negatively regulate ERK signaling pathways during IL-2-induced Jurkat T-cell activation.
